# Enrolling people of color to evaluate a practice intervention: lessons from the shared decision-making for atrial fibrillation (SDM4AFib) trial

**DOI:** 10.1186/s12913-022-08399-z

**Published:** 2022-08-12

**Authors:** Angela Sivly, Haeshik S. Gorr, Derek Gravholt, Megan E. Branda, Mark Linzer, Peter Noseworthy, Ian Hargraves, Marleen Kunneman, Chyke A. Doubeni, Takeki Suzuki, Juan P. Brito, Elizabeth A. Jackson, Bruce Burnett, Mike Wambua, Victor M. Montori, for the Shared Decision-Making for Atrial Fibrillation (SDM4AFib) Trial Investigators, Victor M. Montori, Megan E. Branda, Juan P. Brito, Marleen Kunneman, Ian Hargraves, Angela Sivly, Kirsten Fleming, Bruce Burnett, Mark Linzer, Haeshik Gorr, Elizabeth A. Jackson, Erik Hess, Takeki Suzuki, James IV Hamilton, Peter A. Noseworthy, Alexander Haffke, Jule Muegge, Sara Poplau, Benjamin Simpson, Miamoua Vang, Mike Wambua, Joel Anderson, Emma Behnken, Fernanda Bellolio, Renee Cabalka, Michael Ferrara, Rachel Giblon, Jonathan Inselman, Annie LeBlanc, Alexander Lee, Victor Montori, Marc Olive, Paige Organick, Nilay Shah, Gabriela Spencer-Bonilla, Amy Stier, Anjali Thota, Henry Ting, Derek Vanmeter, Claudia Zeballos-Palacios, Carol Abullarade, Lisa Harvey, Shelly Keune, Timothy Smith, Shannon Stephens, Bryan Barksdale, Theresa Hickey, Roma Peters, Memrie Price, Connie Watson, Douglas Wolfe, Gordon Guyatt, Brian Haynes, George Tomlinson, Paul Daniels, Bernard Gersh, Thomas Jaeger, Robert McBane

**Affiliations:** 1 Knowledge and Evaluation Research Unit, Mayo Clinic, 200 First Street SW, Rochester, MN 55905 USA; 2 Hennepin Healthcare, 730 South 8th Street, Minneapolis, MN 55415 USA; 3 Department of Quantitative Health Sciences, Division of Clinical Trials & Biostatistics, Mayo Clinic, Rochester, MN 55905 USA; 4 Cardiovascular Diseases, Mayo Clinic, Rochester, MN 55905 USA; 5 Department of Biomedical Data Sciences, Leiden University Medical Center, Leiden, The Netherlands; 6 Mayo Clinic Center for Health Equity and Community Engagement Research, Mayo Clinic, Rochester, MN 55905 USA; 7 Indiana University School of Medicine, Indianapolis, IN 46202 USA; 8 Division of Cardiovascular Disease, University of Alabama at Birmingham, Birmingham, AL 32594 USA; 9 Health Partners, Park Nicollet, 8170 33rd Ave S, Bloomington, MN 55425 USA

**Keywords:** Diversity, Minorities, Equity, Enrollment, Practice-based trials, Complex interventions, Shared decision-making, BIPOC

## Abstract

**Background:**

Trial recruitment of Black, indigenous, and people of color (BIPOC) is key for interventions that interact with socioeconomic factors and cultural norms, preferences, and values. We report on our experience enrolling BIPOC participants into a multicenter trial of a shared decision-making intervention about anticoagulation to prevent strokes, in patients with atrial fibrillation (AF).

**Methods:**

We enrolled patients with AF and their clinicians in 5 healthcare systems (three academic medical centers, an urban/suburban community medical center, and a safety-net inner-city medical center) located in three states (Minnesota, Alabama, and Mississippi) in the United States. Clinical encounters were randomized to usual care with or without a shared decision-making tool about anticoagulation.

**Analysis:**

We analyzed BIPOC patient enrollment by site, categorized reasons for non-enrollment, and examined how enrollment of BIPOC patients was promoted across sites.

**Results:**

Of 2247 patients assessed, 922 were enrolled of which 147 (16%) were BIPOC patients. Eligible Black participants were significantly less likely (*p* < .001) to enroll (102, 11%) than trial-eligible White participants (185, 15%). The enrollment rate of BIPOC patients varied by site. The inclusion and prioritization of clinical practices that care for more BIPOC patients contributed to a higher enrollment rate into the trial. Specific efforts to reach BIPOC clinic attendees and prioritize their enrollment had lower yield.

**Conclusions:**

Best practices to optimize the enrollment of BIPOC participants into trials that examined complex and culturally sensitive interventions remain to be developed. This study suggests a high yield from enrolling BIPOC patients from practices that prioritize their care.

**Trial registration:**

ClinicalTrials.gov (NCT02905032).

**Supplementary Information:**

The online version contains supplementary material available at 10.1186/s12913-022-08399-z.

## Introduction

The rigorous evaluation of the value of complex interventions – interventions designed, for example, to improve the organization and delivery of healthcare, the care process itself, or the capabilities of patients or clinicians – often demands the organization and conduct of a randomized trial [1]. The effectiveness of these complex interventions may interact with socioeconomic differences across people and the systems that serve them to produce different treatment effects, which often remain inadequately estimated [2]. These effects must therefore be assessed across a range of socioeconomic circumstances to generate trustworthy estimates of the effect of an intervention, i.e., estimates that are directly relevant and applicable to diverse people and systems [3]. These estimates can then be used to implement interventions that improve quality of care, including equity.

Inequities in the care of BIPOC patients must be corrected by addressing root causes, such as structural racism [4, 5]. Conducting research that can directly apply to the care they receive can also contribute to mitigate and eliminate differences in care experience and outcomes. Yet, the extent to which BIPOC are recruited and enrolled into clinical trials of complex interventions is limited, often as an extended manifestation of these inequities themselves, of their root causes, and of their consequences, e.g., low trust in healthcare [6]. To advance healthcare equity, we need to advance equitable participation in research.

Yet, the organization and conduct of trials to evaluate complex interventions are also under the influence of these same socioeconomic circumstances, affecting the enrollment of people who are underserved, marginalized, traumatized, or distrustful and of the “deep end” practices that provide care to them [7]. Beyond representation, the participation of persons and their practices in the generation of estimates of efficacy contributes to improve trust in their accuracy and may improve the confidence with which the intervention is implemented in practice.

To contribute to the evidence on equitable enrollment in trials of complex interventions, we report here on our team’s experience in promoting equitable participation in a multicenter clinical trial of a shared decision making intervention in patients with atrial fibrillation (AF).

## Methods

The trial protocol was registered on ClinicalTrials.gov (NCT02905032) on 19/09/2016 and has been published elsewhere [8]. In brief, we conducted a multisite randomized trial comparing usual care vs. usual care augmented by clinicians and patients using a shared decision-making tool during the clinical encounter to guide their conversation about the decision to use anticoagulation to prevent strokes in patients with AF. Outcomes of interest were measures of the quality of shared decision making, decisions made and fidelity to their implementation, and cardiovascular health endpoints.

The trial took place in 5 clinics across the United States, three in Minnesota (Mayo Clinic, an academic medical center and lead coordinating site, Park Nicollet Health Partners, an urban/suburban community medical center, and Hennepin Healthcare, a safety-net inner-city medical center), and academic medical centers in Alabama (The University of Alabama at Birmingham Medical Centers) and in Mississippi (University of Mississippi Medical Center). The Mayo Clinic Institutional Review Board and each site’s Institutional Review Board approved all study procedures. Written informed consent was obtained from all participants and all methods were performed in accordance to extant regulations for the conduct of clinical care research. We began enrolling patients in Minnesota in February 2017, with sites serving a more diverse population in Alabama and Mississippi joining later.

Eligible patients were ≥ 18 years of age had nonvalvular AF deemed at high risk of thromboembolic strokes (CHA_2_D_2_-VASc Score ≥ 1 in men, or 2 in women), and had the capacity to provide written informed consent for participation. We excluded patients who were not candidates for anticoagulation or for a discussion about this intervention during the index visit, as determined by their clinician. Study coordinators were required to document each eligibility criterion in the patient’s case report form and kept a log of all patients who were screened and reasons for decline if eligible. The screening log included, when available, the patient’s site of care, age, sex, and race and ethnicity. No waivers or exemptions to any eligibility criterion were permitted.

The main results of this trial have been published elsewhere [9]. The focus of this report is the enrollment of BIPOC participants, either self-identified on a survey question fielded immediately post encounter (by selecting one or more of the following: Black, African American, American Indian, Alaska Native, Asian, Native Hawaiian, Pacific Islander, Hispanic) or, in its absence, abstracted from the medical record. According to the U.S. Census, persons who self-identify with one or more of these groups represent 43.6, 44.7, and 20.9% of the populations of Mississippi, Alabama, and Minnesota, respectively. We aimed at enrolling BIPOC patients to constitute ≥25% of the trial participants.

### Procedures to identify eligible patients

Sites differed in how they identified eligible patients, including the weekly review of scheduled patients with participating or potentially participating clinicians at particular (anticoagulation, general cardiology, internal medicine, electrophysiology) outpatient clinics; clinician referral or electronic alerts for potentially eligible patients, for new diagnosis of AF, or for anticoagulation prescriptions; and the ongoing review of reports of upcoming appointments, emergency visits, and inpatient admissions of patients with AF in their problem list. After a potentially eligible patient was identified, the study coordinator would seek clinician approval to invite the patient into the study, and then approach the patient right before their visit to seek their consent for participation. Only Hennepin Healthcare distributed flyers in the clinic to make patients aware of the study. Clinicians were made aware of the study during formal and informal presentations at staff meetings and via email targeting clinicians likely to see a relatively large number of patients with AF.

### Procedures to optimize BIPOC patient enrollment

One of the study coordinators at Hennepin Healthcare self-identified as African American and another identified as Asian, specifically Hmong, persons. At Mayo Clinic one of the part time study coordinators self-identified as a South Indian person.

Participating site investigators were advised, when possible, to prioritize the enrollment of BIPOC patients with AF. In addition, there were site-specific initiatives, as follows:

- The University of Mississippi site did not change its enrollment processes to prioritize BIPOC participants during the trial.
- At University of Alabama at Birmingham, clinical workflow dictated that only two patients could be recruited per day. To prioritize the enrollment of BIPOC participants, the team shifted its focus from seeking to enroll the first two eligible patients to instead focusing on enrolling the first two BIPOC patients who were eligible to participate.
- At the Park Nicollet and Mayo Clinic sites, the study coordinator continued to screen for eligible patients at all participating clinics electronically but prioritized physical presence at sites more likely to care for BIPOC patients.
- Study coordinators at Hennepin Healthcare sought to recruit patients of Somali origin attending its outpatient clinics. This required the consent form to be translated into Somali and ensuring interpreters were available to initiate study procedures and conduct adequate informed consent processes. Yet, enrollment was hindered by delays in accessing interpreters, the ability of Somali patients to read the written language, and by clinicians’ concerns that the interpreter-assisted clinical encounter would be too lengthy to accommodate the intervention.

### Enrollment logs and site feedback

The process of assessing and enrolling patients was logged at each site. Logs captured patients assessed, whether they were eligibility, whether their clinician had confirmed the eligibility of their encounter, whether the patient was approached for participation, and whether the patient agreed or declined to participate. These logs were periodically submitted to the coordinating center for review and used for feedback and improvement.

Feedback to the sites included reports of rates of enrollment of all and of BIPOC participants at each site over time against targets for enrollment. Strategies to improve the enrollment of participants at each site were reviewed and discussed in formal study meetings and during informal interactions among the study coordinators of all participating sites.

### Statistical analysis

We used descriptive statistics to characterize the patients within each cohort – i.e., enrolled, patient decline, and clinician decline. We tested the significance of difference between cohorts (enrolled-patient decline and enrolled-clinician decline) using t-test, Fisher’s exact test, or chi-squared test as appropriate. To estimate the effect of self-identifying as BIPOC on enrollment (vs. not) we assembled a logistic regression model with enrollment as the dependent variable and age (≥65 vs. < 65), sex, BIPOC or not, and trial site as independent predictors. All statistical analyses were conducted in R. *P*-values were two-sided.

## Results

By the end of recruitment, 2247 people had been assessed for participation in the trial. Of these, 1325 were eligible but did not enroll. Table 1 compares demographic characteristics between eligible participants who did not enroll compared to those who enrolled. Data about race was missing for 5.5% of those eligible but not enrolled compared to 1.5% of those enrolled; data about ethnicity were missing for 8.6 and 3.1% of participants, respectively.

**Table 1:** Patient characteristics

Characteristic	Eligible not enrolled (*N* = 1325)	Eligible, enrolled (*N* = 922)	*P*-value
Age, mean (SD)	71 (11)	71 (10)	0.54^1^
Age, n (%)			0.31^2^
< 50	51 (4%)	27 (3%)	
50–64	286 (23%)	201 (22%)	
≥ 65	922 (73%)	694 (75%)	
Unknown	66	0	
Gender, n (%)			0.81^2^
Female	503 (40%)	363 (39%)	
Male	756 (60%)	559 (61%)	
Unknown	66	0	
BIPOC, n (%)	205 (17%)	147 (16%)	0.99^2^
Race, n (%)			< 0.001^3^
White	1052 (84%)	767 (84%)	
Black	185 (15%)	102 (11%)	
Asian	9 (< 1%)	10 (1%)	
American Indians or Alaska natives	6 (< 1%)	5 (< 1%)	
Native Hawaiian or other Pacific Islanders	0 (0%)	0 (0%)	
Multiple	0 (0%)	22 (2%)	
Other	0 (0%)	2 (< 1%)	
Unknown	73	14	
Ethnicity, n (%)			0.41^2^
Hispanic	5 (< 1%)	7 (< 1%)	
Not Hispanic	1205 (99%)	886 (99%)	
Unknown	115	29	

*BIPOC* Black, Indigenous and people of color; ^1^ – t-test statistic; ^2^ – Chi-squared test statistic; ^3^ – Fisher’s exact test statistic

Only 147 of 922 (16%) enrollees into the trial were BIPOC patients. Although there were no differences in this proportion vs. the proportion of BIPOC eligible participants who did not enroll (*n* = 205; 17%), there were significantly more eligible Black than White participants who did not enroll (185 (15%) vs. 102 (11%); *p* < .001; Table 1).

For those with reasons documented as either patient decline or clinician decline (*n* = 885), the clinician declined the enrollment of the patient-encounter in 632 instances and 253 patients declined participation. Table 2 compares these cohorts against the cohort of eligible patients who enrolled in the trial. Proportionally more eligible BIPOC patients were among those who declined participation than those who enrolled (*n* = 54 (23%) vs. 147 (16%); *p* = .03) while we found no significant difference between the clinician-decline and the enrolled cohorts. This difference is driven by a greater proportion of Black patients among those who declined to participate than among those who chose to enroll (*n* = 47 (20%) vs. 102 (11%); *p* = .001).

**Table 2:** Characteristics of eligible patients by enrollment and by reason for non-enrollment

Characteristic of eligible patients	Not enrolled		Enrolled (*N* = 922)	*P*-value (vs. enrolled)	
	Patient Decline (*N* = 253)	Clinician Decline (*N* = 632)		Patient decline	Clinician decline
Age: Mean (SD)^a^	70 (11)	71 (11)	71 (10)	.38	.96
Age, n (%)^b^					
< 50	11 (5%)	26 (4%)	27 (3%)	.46	.32
50–64	53 (22%)	124 (20%)	201 (22%)		
≥ 65	179 (74%)	456 (75%)	694 (75%)		
Unknown	10	26	0		
Gender, n (%)^b^					
Female	96 (40%)	255 (42%)	363 (39%)	.99	.32
Male	147 (60%)	351 (58%)	559 (61%)		
Unknown	10	26	0		
BIPOC, n (%)^b^	54 (23%)	86 (14%)	147 (16%)	.03	.31
Race, n (%)^c^					
White	190 (79%)	520 (86%)	767 (84%)	.001	<.001
Black	47 (20%)	79 (13%)	102 (11%)		
Asian	2 (< 1%)	5 (< 1%)	10 (1%)		
American Indians or Alaska natives	2 (< 1%)	0	5 (< 1%)		
Native Hawaiian or other Pacific Islanders	0	0	0 (0%)		
Multiple	0	0	22 (2%)		
Other	0	0	2 (< 1%)		
Unknown	12	28	14		
Ethnicity, n (%)^c^					
Hispanic	3 (1%)	2 (< 1%)	7 (< 1%)	.44	.33
Not Hispanic	228 (99%)	594 (99%)	886 (99%)		
Unknown	22	36	29		

*BIPOC* Black, Indigenous and people of color; ^a^ – t-test statistic; ^b^ – Chi-squared test statistic; ^c^ – Fisher’s exact test statistic

**Fig. 1:**
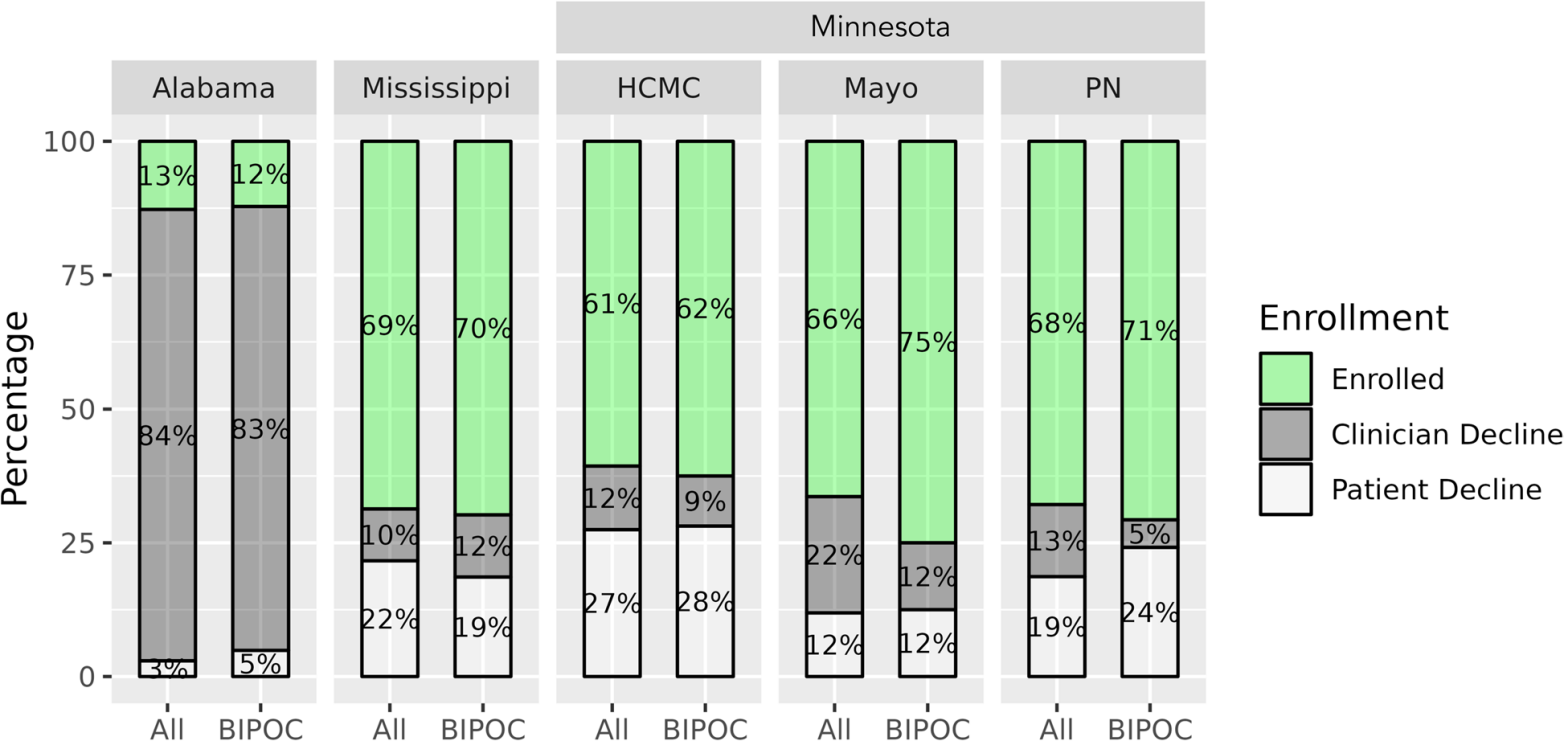
Proportion of BIPOC participants enrolled, by participating trial site. Proportion of Black, Indigenous and people of color (BIPOC) patients by enrollment status and main reasons for non-enrollment. Total enrollment of BIPOC patients was 147, 10 from University of Alabama at Birmingham (AL), 60 from Hennepin Healthcare (HCMC), 6 from Mayo Clinic, 30 from University of Mississippi (MS), and 41 from Park Nicollet (PN)

Figure 1 shows the distribution of enrolled and not enrolled (clinician decline and patient decline) across sites by all participants and BIPOC. While demonstrating different policies at each site (e.g., University of Alabama’s process led to more instances of clinicians declining the enrollment of encounters overall and with BIPOC population than at other sites), we found no major differences within site between the enrollment and decline rates in the whole participant cohort vs. the BIPOC cohort.

The supplemental table reports the number of participants across the cohorts by BIPOC status and by site.

While patient age, gender, and race were not significant predictors of enrollment, study site significantly predicted enrollment in the logistic regression model (*p* < .01).

**Fig. 2:**
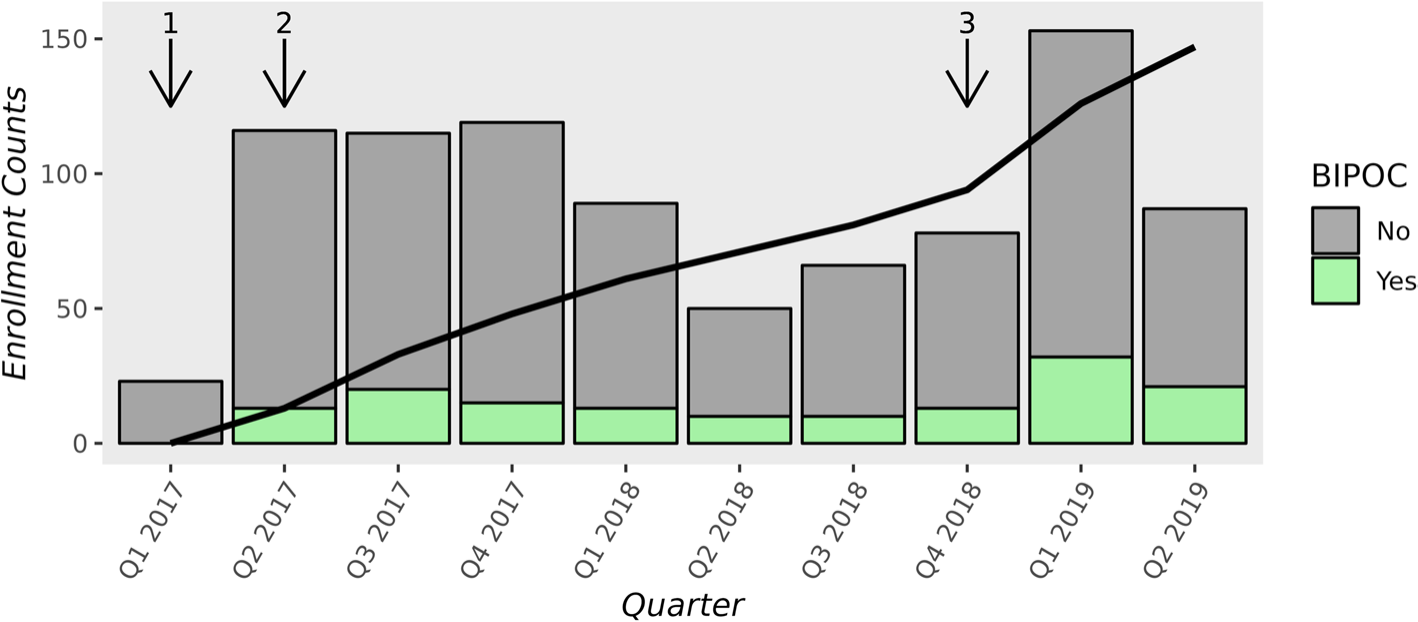
Enrollment over time. The black line represents the cumulative enrollment of Black, Indigenous and people of color (BIPOC) patients. The arrows depict the start of enrollment at participating health systems: (1) Mayo Clinic (first quarter (Q1), 2017); (2) Hennepin Healthcare and Park Nicollet (Q2, 2017); and (3) University of Alabama at Birmingham and University of Mississippi (Q4, 2018)

Figure 2 shows participant enrollment over time by BIPOC status, demonstrating the effect of incorporating two sites that serve a higher proportion of Black patients on BIPOC enrollment rates.

## Discussion

The inclusion and prioritization of clinical practices that care for more BIPOC patients contributed to a higher enrollment rate into a randomized trial to evaluate a complex intervention in clinical practice. Specific efforts to reach BIPOC clinic attendees and prioritize their enrollment had lower yield. Overall, only 1 in 6 enrollees were BIPOC, almost all of them Black.

To some extent, these findings can be explained by the proportion of eligible Black participants who declined to participate, rather than by clinicians’ decisions to decline enrollment of patient-encounters. However, the racial composition of the population able to access and receive care at participating sites seems the key determinant, with a noticeable inflection in the number of Black patients enrolled into the trial as sites in Alabama and Mississippi began enrolling (Fig. 2). According to the annually updated U.S. Census American Community Survey, 50.5% of the population in Jefferson County, Alabama and 75.1% of the population in Hinds County, Mississippi use a race or ethnicity descriptor other than White.

On the other hand, efforts to prioritize the enrollment of BIPOC participants may have had an important impact that differed across sites. BIPOC patients represented 16 and 32% of participants assessed for inclusion at Alabama and Mississippi, respectively. Conversely, BIPOC patients represented 39% of all patients assessed for enrollment at Hennepin Healthcare while the population at Hennepin County, Minnesota that self-described using a category other than White approached 32%. This suggests that the presence of BIPOC study coordinators and the prioritization, within that healthcare system, of practices that care for more diverse populations, may have been effective. Yet other barriers remained. Investigators at Hennepin Healthcare decided to focus enrollment on Somali patients, who constitute 1% of the population of Minnesota [10]. Trial enrollment required the participation of an interpreter, as few patients could read the consent form. There were insufficient funds to translate study material and hire a dedicated interpreter. Clinicians were concerned that there would not be enough time to wait for and work with the interpreter to complete study procedures. As a result, only a few Somali patients were deemed eligible for the study and none were enrolled.

The first and rather obvious conclusion of our study is that recruitment in practice-based trials will reflect the racial composition of the population affected by the condition of interest (the prevalence and incidence of atrial fibrillation is reportedly lower in Black patients [11, 12]) served by the practice, which in turn reflects the underlying demographics minus the effects of racist policies on access to care (e.g., practice location, affordability, staff diversity, practice policies). Thus, to generate trustworthy estimates of the effect of complex interventions in clinical practice that are applicable to BIPOC populations, trialists must prioritize the participation of practice sites that have implemented policies that favor the care of BIPOC populations. Practices that seek to serve this population would have a racially diverse clinical staff and would have implemented policies – convenient location and accessibility, longer and more accommodating appointment times, point-of-care interpreters, and other policies designed to serve BIPOC populations in a minimally disruptive manner.

A key limitation of our evaluation is that we know neither the race/ethnicity of participating clinicians nor the extent to which this shaped enrollment of BIPOC patients. Also, the practical nature of our trial limited the data that could be collected. We did not document detailed reasons for clinicians to decline the enrollment of their eligible patients or for patients to decline participation.

In addition to simplifying eligibility criteria, trial approaches worthy of further study include the employment of BIPOC study coordinators and the design of trial procedures with input of community members for the population of focus through community-engaged processes. Other approaches include tabling (have a table in public locations staffed by racially concordant research staff to enroll patients), community outreach, and partnering with BIPOC community leaders and businesses, churches, and other community organizations [13]. While typically recommended to improve the enrollment of BIPOC populations in clinical trials, these approaches apply to trials that recruit participants directly and were not available to us and are generally less applicable to practice-based trials mediated by clinicians and healthcare services. For these trials, however, it may be more effective to modify trial funding and allocation of funds to specific sites caring for BIPOC patients.

In addition to advocacy to promote equity, diversity, and inclusion of research and clinical staff and of the patients served, there is also ample opportunity to develop the evidence for the effectiveness of interventions designed to optimize the enrollment of BIPOC participants into practice-based trials. To this end, it may be important to collect site-specific demographics, evaluate the need for additional time, if needed, to include all potential enrollees, and efforts to better understand why clinicians do not include all eligible participants.

In conclusion, a substantial opportunity remains to identify best practices to optimize the enrollment of BIPOC participants into practice-based trials that examine complex interventions. This study suggests a high yield from enrolling BIPOC patients from practices that prioritize their care. This approach and other inclusive designs may improve the representation of BIPOC populations and the applicability of study findings to their care.

## Supplementary Information

**Additional file 1 Supplemental Table A.** Patients by health system grouped by enrollment.

## Data Availability

The datasets generated and/or analysed during the current study are not currently publicly available but they are being prepared in adherence to a data sharing agreement with the National Institutes of Health. In the meantime, these are available from the corresponding author on request.
