# Supplementary material for: Enrolling people of color to evaluate a practice intervention: lessons from the shared decision-making for atrial fibrillation (SDM4AFib) trial
Source: BMC Health Serv Res. 2022 Aug 12;22:1032. doi: 10.1186/s12913-022-08399-z (PMC9375357; doi:10.1186/s12913-022-08399-z)
Supplement: Supplementary file 1 — Additional file 1 Supplemental Table A. Patients by health system grouped by enrollment. [file 12913_2022_8399_MOESM1_ESM.docx]

**Supplemental Table A: Patients by health system grouped by enrollment**

|  |  | **Recruitment healthcare system**  **N (% of total per system)** | | | | |
| --- | --- | --- | --- | --- | --- | --- |
| **Status** | **Group** | University of Alabama | Hennepin Healthcare | Mayo Clinic | University of Mississippi | Park Nicollet |
| Enrolled | BIPOC | 10 (15.4) | 60 (40.5) | 6 (2.1) | 30 (32.6) | 41 (12.5) |
|  | White | 53 | 88 | 262 | 62 | 284 |
|  | Missing | 2 | 0 | 22 | 0 | 2 |
|  | **Total** | 65 | 148 | 290 | 92 | 327 |
| Clinician Decline | BIPOC | 68 (16.2) | 9 (31) | 1 (1.1) | 5 (38.5) | 3 (4.6) |
|  | White | 362 | 18 | 65 | 8 | 62 |
|  | Missing | 0 | 2 | 29 | 0 | 0 |
|  | **Total** | 420 | 29 | 95 | 13 | 65 |
| Patient Decline | BIPOC | 4 (26.7) | 27 (40.3) | 1 (1.9) | 8 (27.6) | 14 (15.6) |
|  | White | 11 | 39 | 38 | 21 | 76 |
|  | Missing | 0 | 1 | 13 | 0 | 0 |
|  | **Total** | 15 | 67 | 52 | 29 | 90 |

BIPOC: Black, Indigenous and people of color
